# Wood Bottom Ash and GeoSilex: A By-Product of the Acetylene Industry as Alternative Raw Materials in Calcium Silicate Units

**DOI:** 10.3390/ma13020489

**Published:** 2020-01-20

**Authors:** Manuel Angel Felipe-Sesé, Luis Pérez-Villarejo, Eulogio Castro, Dolores Eliche-Quesada

**Affiliations:** 1 Department of Chemical, Environmental and Materials Engineering, Higher Polytechnic School of Jaén, University of Jaen, Campus Las Lagunillas s/n, 23071 Jaén, Spain; mfelipesese@gmail.com (M.A.F.-S.); ecastro@ujaen.es (E.C.); 2 International University of La Rioja, Gran Vía Rey Juan Carlos I, 41, 26002 Logroño (La Rioja), Spain; 3 Department of Chemical, Environmental, and Materials Engineering, Higher Polytechnic School of Linares, University of Jaen, Campus Científico-Tecnológico, Cinturón Sur s/n, 23700 Linares (Jaén), Spain; lperezvi@ujaen.es; 4 Center for Advanced Studies in Earth Sciences, Energy and Environment (CEACTEMA), Universidad de Jaén, Campus Las Lagunillas, s/n, 23071 Jaén, Spain

**Keywords:** biomass bottom ash, GeoSilex, calcium silicate units, mechanical and thermal properties, sustainability

## Abstract

The main objective of this research was to obtain calcium silicate units from alternative raw materials, such as the bottom ashes from the combustion of wooden boards (WBA), as a source of silica, and GeoSilex (G), a by-product with low energy and environmental costs generated in the manufacture of acetylene, as a source of lime. Once the raw materials were physically, mineralogically and chemically characterized, calcium silicate units were obtained by mixing different amounts of WBA residue (90–20 wt%) and G by-product (10–80 wt%). The mixtures were compressed at 10 MPa and cured in water for 28 days. The calcium silicate units were subjected to a wide experimental program that included the determination of physical properties (bulk density, apparent porosity and water absorption), mechanical properties (compressive strength), and thermal properties (thermal conductivity). Optimum values are obtained for calcium silicate units that contain a 1/1 WBA/G weight ratio, which have an optimal amount of SiO_2_ and CaO for the cementation reaction. The 50WBA-50g units have compressive strength values of 46.9 MPa and a thermal conductivity value of 0.40 W/mK. However, all calcium silicate units obtained comply with the European Standard EN 771-2: 2011 to be used as structural building materials.

## 1. Introduction

In 2016, the total amount of wastes produced in the European Union (EU) by the entire economic activities and households reached 2538 million tons; this represented the largest amount recorded during the period 2004–2016. Approximately more than half of these wastes (53.5%) were treated in recovery operations: recycling (37.5%), backfilling (10.1%) or energy recovery (5.6%). The rest of the recovery waste (46.5%) was either incinerated without energy recovery (1.0%) or disposed in landfills (45.5%) [1]. Globally, approximately 1.3 billion tons of waste are generated per year and the volume is expected to increase up to approximately 2.2 billion tons per year by 2025 [2].

One key point to the circular economy is turning waste into a resource that can be used in an efficient and sustainable way. The improvement in waste management helps to reduce environmental and health concerns, mitigating greenhouse gas emissions and avoiding negative impacts such as local landscape deterioration and water and air pollution. The EU approach to waste management established the following priority order in the 7th Environment Action Programme, which will lead European environment policy until 2020: prevention, reuse, recycling, recovery and, as the least preferable option, landfilling [3].

Some wastes derived from organic material, such as plant, trees, and agricultural and urban waste, can be transformed into energy through combustion to generate heat and/or electricity. These wastes constitute what is called biomass, which has been proved to be extremely effective (when is produced in a sustainable way) at reducing greenhouse gas emissions. Around 64% of total primary energy production of renewable energy in the EU in 2016 was generated by this ‘indirect solar fuel’ source [4].

Energy production from biomass has important environmental advantages; however, it also has the drawback of the large quantities of ashes generated as a result of the combustion process, which are usually transported to landfills for deposition, causing growing environmental problems. It must be considered that global ashes production from biomass is currently estimated over 480 Mtons/year [5,6]. Bottom and fly ash are generated in the combustion [7]. Biomass bottom ash (BBA), is produced in the furnace, includes the coarse fraction and is formed by the total or partially burnt material [8]. It is composed of mineral impurities mixed with sand particles [9]. It is composed of mineral particles mixed with sand particles. Biomass fly ash (BFA) is separated by filters from the stream of gases outside the combustion chamber, so it is the finest and lightest fraction of the ashes [10]. BBA has a minor organic fraction and mainly inorganic fraction [11]. In Spain, power production plants using biomass as fuel generate 120,000 tons/year of wastes [12], of which approximately 64% are BBA and 36% BFA [13].

Appropriate waste management strategies for biomass ash (BA) are needed to promote the sustainability of energy production from this material. The quantity and quality of the burnt waste in the form of ashes resulting from the biomass combustion are directly related to the characteristics of the biomass employed, in combination with the technology and operating conditions of the combustion process [6,14,15,16,17,18]. Different studies have been carried out to investigate the applications of this waste, which can be classified into two groups in accordance with the potential of this material: on the one hand, as a fertilizer and stabilizer on soils in forestry and agriculture and, on the other hand as a raw material in building products and civil engineering [19,20,21].

Though BA has proven its potential uses, this residue is still often disposed of in landfills due to regulatory barriers along with the current concerns about the possible leaching of contaminants to soil ecosystems. In this regard, a research evaluating the potential applications of different BA in soil and cement mortars has been recently conducted [22]. The study concluded that an approach based on potential leachability instead of the elements contained in ashes provides a more realistic assessment of the availability of nutrients and contaminants, giving rise to an incentive for the sustainable use of BA as soil amendment. On the other hand, in this research, samples of mortars were produced replacing cement with BBA (20–40 wt%) and it was shown that mortars complied with strength requirements and environmental leaching criteria for construction products, supporting the possibility that the beneficial utilization of biomass ash for building purposes might be more feasible than direct soil application.

The use of BA as partial replacement for cementitious materials in construction materials is dependent by its physical-mechanical properties. BFA has been more broadly studied due to its outstanding binding properties; however, there are fewer studies on the use and management of BBA. It has been proven that the use of certain BBA in the manufacture of mortar and concretes could be also feasible due to the high content in CaO and SiO_2_; however, it also could have some limitations due to the content of NaO_2_ and KO_2_, which might causes shrinkage and deformation problems in the matrix [11]. Other factors to consider are the habitually higher presence of organic matter in BBA compared to BFA and the particle size—BBA has maximum nominal sizes of 2–9 mm, whereas BFA particle size has a maximum of 200 µm [11]. Likewise, there are significant differences in the crystalline-amorphous present phases of both type of ashes [23,24,25,26].

Notwithstanding the preceding, several studies recently conducted have demonstrated the usefulness of BBA in certain applications, such as in road structural layers to improve the bearing capacity with satisfactory results [27,28,29], as a cement substitute in concrete and cement mortars [30,31,32,33], obtaining an improvement in the mechanical and durability, as raw material in cement panels [34], and in clay fired bricks [35,36]. It should be noted that BBA from agro-industrial by-products has a high percentage of silica and as a consequence a potential cementitious capacity for its use in cement-treated materials [37].

The manufacture of calcium silicate masonry units using alternative raw materials to conventional ones has been studied by other authors. The following are some examples: bottom ash from the combustion of a mix of orujillo and energy crops in combination with different sources of lime [38]; blast furnace slag together with hydraulic limes [39]; limestone powder along with class C fly ash and silica fume [40]; hospital waste incineration bottom ash with a class 42.5 R ordinary Portland cement (OPC) [41]; water-based drilling cuttings (WDC) from oil and gas field with cement and fine aggregates [42]; and coal fly ash waste combined with GeoSilex [43]. In all of this research, the optimal quantities of raw materials, together with the appropriate conformation and curing conditions, have been studied in order to produce masonry units with adequate technological properties.

Building on these antecedents, the use of wood bottom ash (WBA) resulting from the combustion process of the residues generated in the wooden board manufacturing industry has been studied in the present work with the intention of producing new sustainable bricks. This type of biomass material is constituted primarily of wood scraps from conifer bark (52%), wood dust, and nonconforming products (48%) and generates, as a result of its combustion, ashes with a high content in oxides, primarily silica. These ashes are generated in the furnace of a cogeneration plant to obtain electrical and thermal energy and they are currently accumulating in authorized dumps.

The present study is particularly relevant, due to the added value of using another residue called “GeoSilex” in combination with the preceding to achieve the objective pursued. This by-product, is basically constituted by hydrated lime [Ca(OH)_2_] and it is generated by acetylene industry as a product of the following reaction: CaC_2_ + 2H_2_O → Ca(OH)_2_ + C_2_H_2_. At present, this material has become a raw material with potential applications after the removal of impurities such as sulphates, sulphites, sulphides, and organic carbon. The main component of GeoSilex is nano-portlandite (85%) and is currently used as an additive in the ceramic industry [43]. This study attempts to verify the feasibility of using these alternative raw materials, which have never been used in combination, for the production of unfired bricks.

As a result, this research can contribute to the sustainability of the construction industry by reducing the consumption of natural resources, such as sand, through the use of wastes and by-products, which result to be efficient materials. Likewise, it should be taken into account that conventional calcium sources, such as quicklime and Portland cement are obtained by calcination at high temperatures generating significant CO_2_ emissions. In contrast, the by-product GeoSilex, is a by-product of the acetylene industry, manufactured entirely from waste. For this reason, GeoSilex is a positive environmental action reagent due to its high capacity to absorb environmental CO2 and its almost zero energy consumption

In addition, as is well known, the production of masonry bricks requires additional energy due to firing or steam curing of the samples. However, in this study the curing process is carried out in water at room temperature, with the consequent, energetic saving and economic and environmental benefits.

The present study aims at providing further knowledge in the properties and physical, mechanical and thermal behavior of eight different calcium silicate units made using different amounts of biomass bottom ash (90–20 wt%), as silica source, and GeoSilex (10–80 wt%), as calcium oxide source, with the intention of evaluating the feasibility of using these residues, as raw materials for the development of unfired sustainable calcium silicate units.

## 2. Materials and Methods

### 2.1. Raw Materials

The raw materials used were wood bottom ash (WBA) as a silica source, and GeoSilex (G) as a lime source for the manufacturing of calcium silicate units.

**Figure 1:**
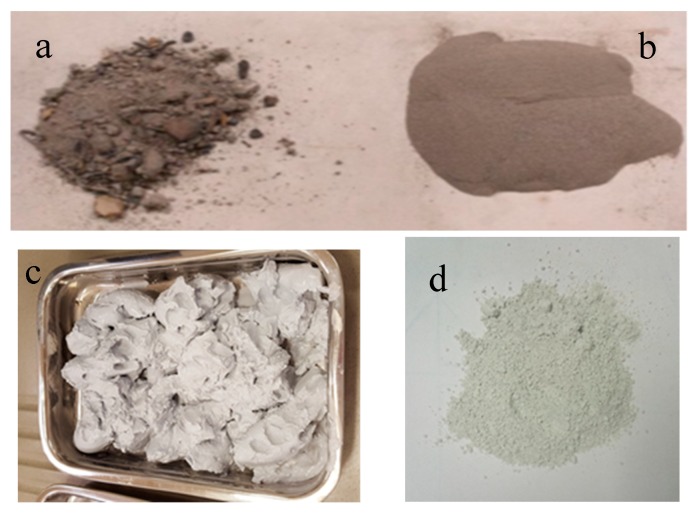
Pictures of raw materials (**a**) raw wood bottom ash (WBA); (**b**) WBA after sieving; (**c**) GeoSilex (G) paste; and (**d**) G powder.

WBA were supplied by the factory Tableros Tradema S.L. located in Linares (Jaén, Spain). This company uses in its facilities by-products from the processing of wooden boards as fuel. This material had heterogeneous particles size and included metal scraps. (Figure 1a). The metal scraps were removed. Subsequently the ashes were crushed into an agate ball mill. Finally, the ashes were sifted with a sieve of mesh size of 150 µm (Figure 1b).

GeoSilex (G) has been used as a source of lime. This material is obtained as waste in the acetylene industry. GeoSilex by-product was supplied as a paste (Figure 1c) by the Company GeoSilex Trenza Metal S.L (Zaragoza, Spain). First, moisture is removed by drying in an oven at 80 °C. After drying, the by-product was ground in a ball mill to obtain an homogeneous particle size (Figure 1d).

### 2.2. Characterization of Raw Materials

A PCE-PH20S pH meter of PCE instruments was used to determine the pH of the raw materials. ASTM D-2974 standard [44] was used to determine the organic matter content. Carbonate content has been determined by Bernard calcimetry. A volumetric flask (Le Chatelier, Pobel, Madrid, Spain) has been used to determine the relative density. Blaine Air-Permeability equipment [45] was employed to determine the specific surface area.

Particle size distribution of raw materials was measured using a Malvern Mastersizer 2000 laser diffractometer.

A Philips Magix Pro X-Ray Fluorescence (XRF, Philips/PAnalytical, Malvern, UK) equipment model PW-2440 was used to determine the chemical composition of the raw materials.

A PANalytical X′Pert Pro MPD automated diffractometer with Ge (111) primary monocromator using 40 mA, 45 kV and CuKα radiation coupled with an X′Celerator detector was used to determine the crystalline phases.

### 2.3. Preparation of Wood Bottom Ash-GeoSilex Calcium Silicate Units

**Figure 2:**
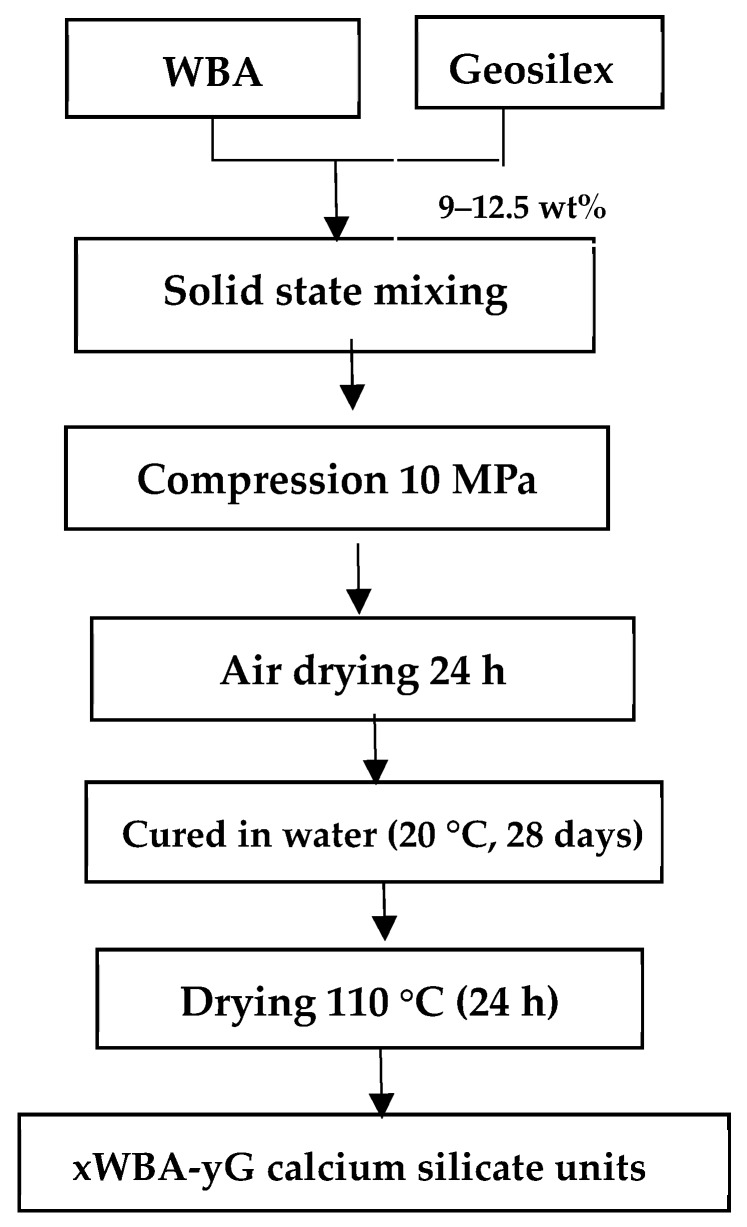
Flowchart of producing calcium silicate units using as raw materials wood bottom ash (WBA) and GeoSilex (G).

Calcium silicate units were manufactured mixing wood bottom ash (WBA) and GeoSilex (G) as cementitious agent. Suitable amounts of raw materials were mixed for 5 minutes in solid state using a Proeti planetary kneader. The necessary amount of water, 9–12.5 wt%, was then added so that the mixture obtained the appropriate plasticity for the subsequent stage of compression in a semi-dry state. Eight series of samples denoted xWBA-yG were prepared where x indicates wt% in WBA (90–20 wt%) and y indicates wt% in G (10–80 wt%) (Table 1). Ten specimens of each sample with an individual weight of 40 g and dimensions of 60 × 30 × 15 mm were manufactured. The samples were shaped cylindrically by applying a 10 MPa uniaxial load for 60 s. Subsequently, moisture was removed from the green pieces by drying at 105 °C (24 h). The specimens were then cured in water (28 days at room temperature) according to UNE-EN 12390-2 standard [46]. Finally, the calcium silicate units were dried until constant weighing (Figure 2).

### 2.4. Characterization of Calcium-Silicate Units

The physical, mechanical and thermal properties of the calcium silicate units were determined in accordance with the standards. The UNE-EN 772-16 standard [47] was used to determine the dimensions of shaped units. The Archimedes method was followed to determine bulk density. The water absorption was obtained on the basis of the UNE-EN 772-21 standard [48]. A MTS 810 Material Testing Systems laboratory press was used to obtain the compressive strength of specimens according to UNE-EN 772-1 standard [49]. Thermal conductivity of the units at 10 °C was performed with a FOX 50 TA Instruments heat flow meter in accordance with the standard ISO 8302: 1991 [50]. 

The Fourier-transform infrared spectroscopy (FTIR) spectra of calcium silicate units were obtained using a FTIR Bruker Tensor 27 equipment. The total attenuated reflection method (ATR) was used. The spectra were obtained between 4000–400 cm^−1^ using a resolution of 4 cm^−1^ in the absorbance mode.

Scanning Electron Microscopy and Energy Dispersive X-ray Spectroscopy (SEM/EDS) was performed with a high-resolution transmission electron microscope, model JEOL SM 840, combined with EDS chemical analysis (20 kV) to evaluate the microstructure of calcium silicate units. A ion sputtering device JEOL, model JFC 1100, was used to coat the specimens with carbon.

## 3. Results and Discussion

### 3.1. Characterization of Raw Materials

The organic matter content of the WBA residue is high (Table 2), indicating an inefficient combustion of the wood. The carbonate content of the two raw materials is high. The high carbonate content of the by-product GeoSilex is due to its easy carbonation. The pH of the raw materials is basic, that favors the pozzolanic reaction of the silica of the wood bottom ash and the lime contained in the GeoSilex. The specific surface area values of the residue and the by-product obtained by the Blaine method indicate that the by-product G has a greater surface area, which performs a favorable action on reactivity, indicating a possible greater reactivity of the G by-product than the WBA residue. The bulk density of the WBA is higher than that obtained for G.

**Figure 3:**
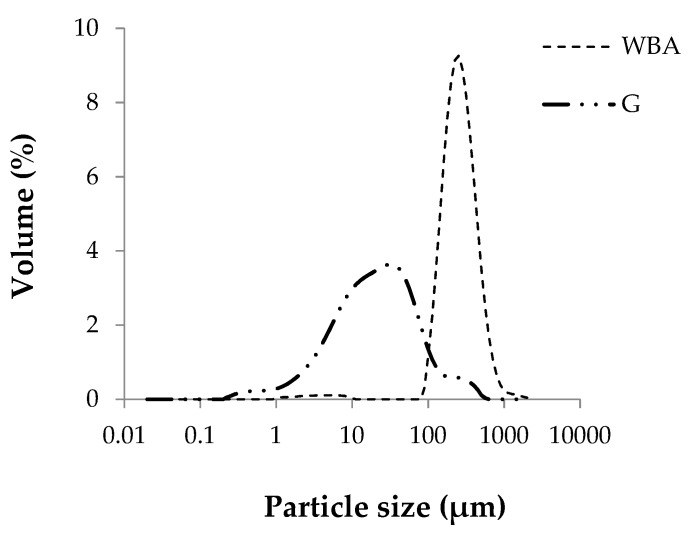
Particle size distribution of raw materials wood bottom ash (WBA) and GeoSilex (G).

Figure 3 shows the particle size distribution of raw materials. The average particle size, D_50_, of the GeoSilex by-product is smaller than that of the WBA residue, being 21.8 μm for the G, increasing to 237.6 μm for the ash residue. WBA particles present most of their particles (98.3%) in the size range to the sand (0.063–2 mm). However, the G by-product has a higher percentage of particles (74.68%) of silt size (0.002–0.063 mm). This result indicates that G, having a smaller particle size, would have higher pozzolanic activity due to the greater surface area of the particles and the increase in the rate of hydraulic reaction [51].

The chemical composition of the WBA and G raw materials determined by XRF (Table 3) indicates that the WBA residue consists mainly of silica (48.6%), followed by calcium oxide (18.1%) and alumina (5.9%). The content of oxides of iron, magnesium, titanium and potassium is between 1% and 4%.

The by-product GeoSilex is mainly composed of calcium oxide (67.2%). Due to its easy carbonation, G has a high loss on ignition (LOI: 27.7%). The WBA residue due to its high silica content can be used as a pozzolanic material, which requires the activator G by-product, rich in calcium hydroxide, which facilitates the initiation of the cementation reaction.

**Figure 4:**
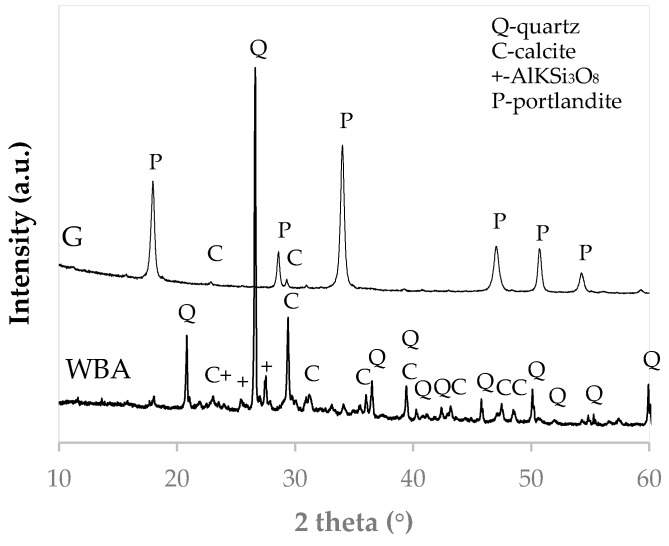
XRD pattern of WBA residue and G by-product.

The WBA X-ray diffraction (XRD) pattern (Figure 4) shows that the main crystalline mineralogical phase of the residue is quartz. It also has lower intensity diffraction peaks corresponding to calcium carbonate (CaCO_3_) and aluminum and iron silicates (AlKSi_3_O_8_). The XRD pattern of the by-product GeoSilex indicates that it is mainly constituted of portlandite (Ca(OH)_2_), due to the easy carbonation of the by-product, with small diffraction peaks corresponding to calcium carbonate.

### 3.2. Characterization of the Calcium-Silicate Units

**Figure 5:**
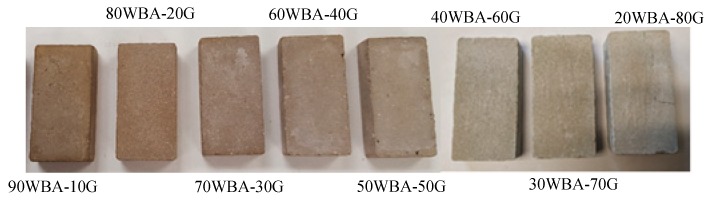
Calcium silicate units.

Figure 5 shows the calcium-silicate units after 28 days of curing in water. The specimens showed no defects such as cracks. The color of the specimens is brown, being clearer as greater additions of G by-product were incorporated.

The presence of efflorescence is slightly observed in specimens containing 70 wt% of WBA or lower. This defect is due to crystallization of soluble salts, contained in the raw materials used in the manufacturing process of calcium silicate units. During the curing process, water-soluble salts can be transported by capillarity through the porous materials and deposited on its surface when it evaporates. Normally it is a slight problem of aesthetic type, which does not affect the durability of the units.

**Figure 6:**
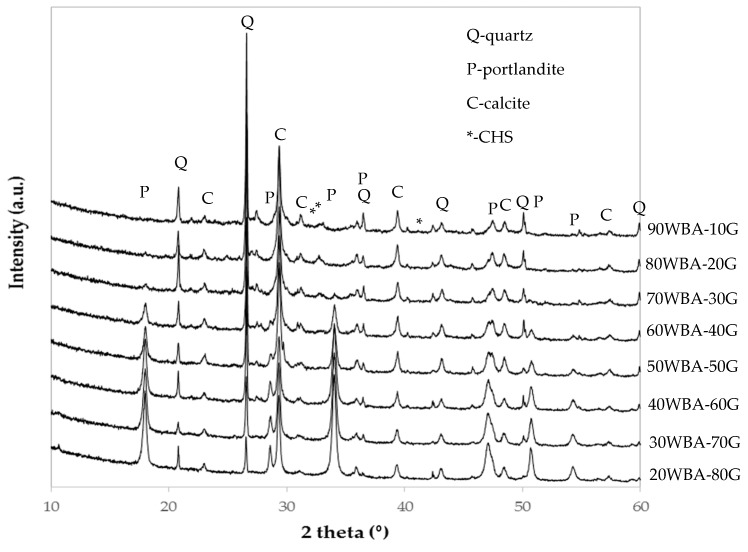
XRD patterns of calcium silicate units.

The XRD patterns of the calcium-silicate units (Figure 6), indicate the presence of diffraction peaks corresponding to unreacted raw materials species, such as quartz present in the WBA residue, portlandite present in the G by-product and calcite present in both raw materials. The quartz peak is more intense as increasing quantities of ash were incorporated, while the portlandite peak intensifies as increasing amounts of GeoSilex were incorporated. In addition, small diffraction peaks corresponding to hydrated calcium silicates due to their amorphous character, can be observed. These diffraction peaks are less intense as increasing amounts of GeoSilex (60–80 wt%) were incorporated, which could indicate an excess of calcium oxide and an insufficient amount of silica and alumina present. This fact could lead to a lesser extent of the pozzolanic reaction.

**Figure 7:**
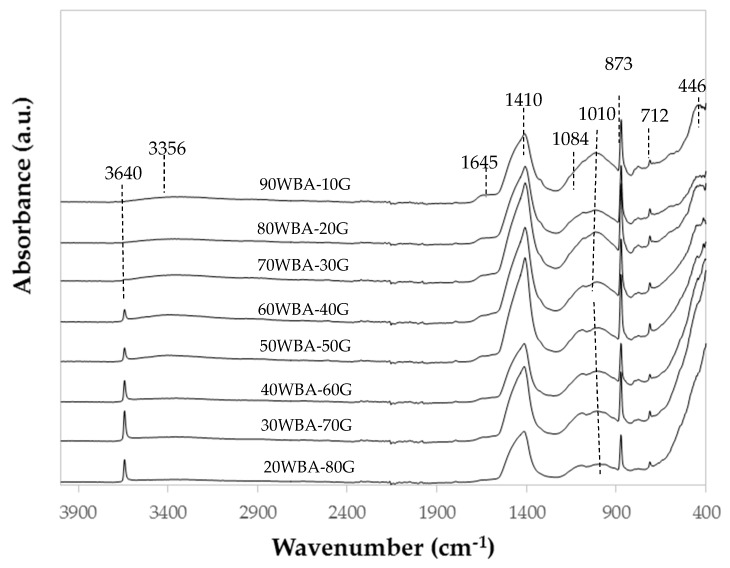
FTIR spectra of calcium silicate units.

Different regions can be distinguished in the Fourier-Transform Infrared Spectroscopy (FTIR) spectra of the calcium silicate units (Figure 7). In the region of 3800–3000 cm^−1^ a wide band centered at 3381–3346 cm^−1^ is observed, whose intensity decreases when GeoSilex raw material content increases. This band can be assigned with the stretching vibration of the water molecules present in the hydrated calcium silicates formed during the hydration reaction of raw materials, WBA and G [52,53,54]. In FTIR spectra, GeoSilex percentages greater than 50 wt%, a peak centered at approximately 3640 cm-1 is also observed, which may be due to the stretching vibration of υ (OH) of unreacted Ca(OH)_2_ present in the G by-product [54].

In the region between 1800 and 1200 cm^−1^, a small band centered at 1645 cm^−1^ can be observed whose intensity decreases as increasing amounts of GeoSilex are incorporated. This band can be due to the bending modes of the water molecules present in the CSH gel [52,53,54]. In addition, a more intense band centered at approximately 1408–1413 cm^−1^ is observed. This band can be attributed to the vibration modes of (CO_3_)^2−^ present in raw materials [53,54,55].

In the region between 1200–750 °C, an intense double band centered at 1084–1089 cm^−1^ and 1111–999 cm^−1^ can be observed. The first band is characteristic of the stretching modes of Si-O present in unreacted ashes. The second band is characteristic of the stretching modes of Si–O present in hydrated calcium silicates formed in the reaction pozzolanic [55,56,57,58]. This band shifts to lower wavenumbers to which up to 50 wt% of G by-product is incorporated. Higher additions (60–80 wt%) produce a shift towards higher wavenumbers. This event indicates a greater amount of hydrated calcium silicates in 50WBA-50g calcium silicate units The intense peak centered at 873 cm^−1^ and the least intense centered at 712 cm^−1^ are assigned to the vibrations of the (CO_3_)^2−^ group [59].

The bands at 446 cm^−1^, which more intense and with a greater amount of WBA residue in the specimens, could be due to the vibration of stretching Si–O and Al–O of the silicate and aluminate groups [58].

**Figure 8:**
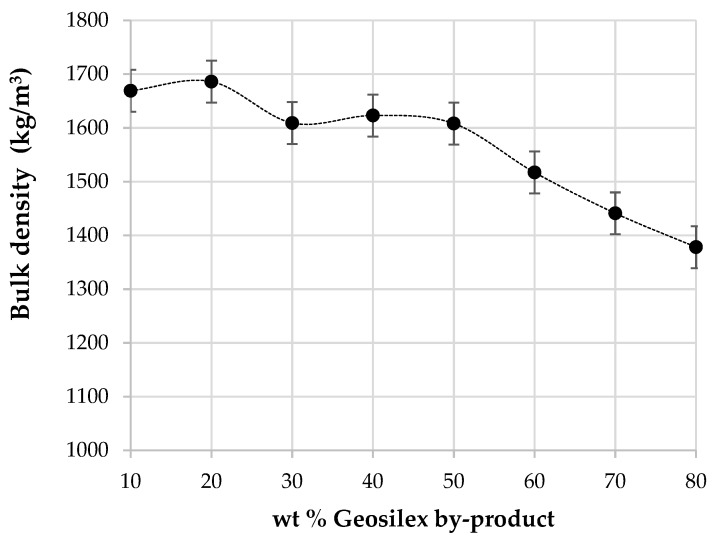
Bulk density of the xWBA-yG calcium silicate units as function of G by-product content.

The bulk density of the calcium silicate units as a function of the amount of G by-product added as pozzolanic material is shown in Figure 8. The bulk density for specimens with higher WBA residue content (90WBA-10g) is 1669 kg/m^3^ decreasing to 1378 kg/m^3^ with the addition of 80 wt% of G by-product (20WBA-80g). It is observed that, in general, bulk density decreases as increasing amounts of G are incorporated. The relative density of the WBA residue is 2731 kg/m^3^, higher than those obtained for G by-product, 2378 kg/m^3^. Therefore, the incorporation of G results in calcium silicate units with lower bulk density and greater porosity. However, calcium silicate units with G content of 40–50 wt% (60WBA-40G and 50WBA-50G) have similar bulk densities of 70WBA–30G, despite the increase in G of lower relative density. This may be due to the fact that these specimens have adequate amounts of ash and GeoSilex, which in the cementing reaction results in a greater amount of calcium silicates hydrated in these calcium silicate units, which give rise to a more compact structure and less porosity. The increase in bulk density when the ash content increases has been described in other studies. Eliche-Quesada et al. [43], indicated bulk densities between 1481 kg/m^3^ and 1341 kg/m^3^ for specimens containing between 80 wt% of coal fly ash and 20 wt% GeoSilex and 30 wt% of coal fly ash and 70 wt% of GeoSilex. Carrasco-Hurtado et al. [38] obtain similar values of bulk density, between 1584–1333 kg/m^3^ when bottom ashes of a mix of orujillo and energy crops and, calcium oxide or calcium hydroxide, as source lime, are used. The use of Portland cement increases the bulk density up to 2037 and 1757 kg/m^3^. Similar values of bulk density (1790 kg/m^3^) are obtained by Turgut [40] for masonry bricks using as raw materials limestone powder, class C fly ash, and different content of silica fume (0–20 wt%).

**Figure 9:**
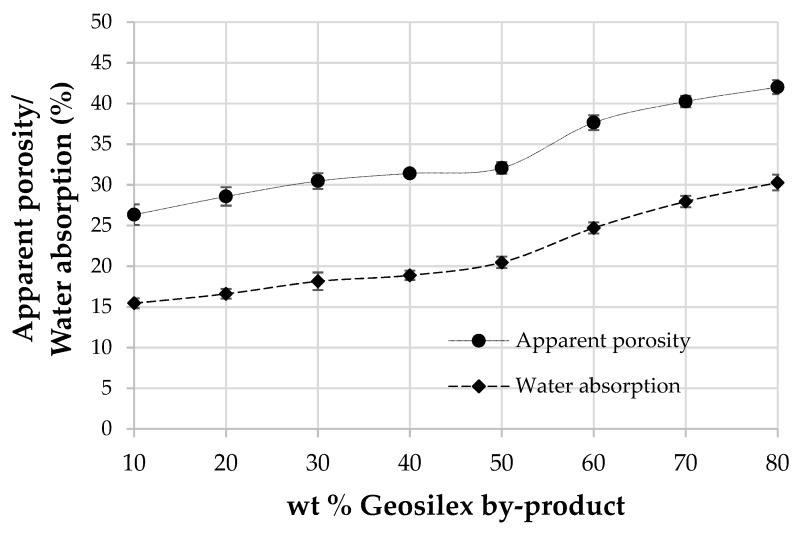
Apparent porosity and water absorption of the xWBA-yG calcium silicate units as function of G by-product content.

The apparent porosity and water absorption of calcium silicate units as a function of the G by-product content is shown in Figure 9. The porosity of specimens makes them vulnerable to external effects (rain, pollutants) and chemical effects [60] while water absorption, an indirect measure of open porosity, affects the specimen’s durability. The apparent porosity and water absorption is incremented as increasing amounts of the G by-product are incorporated. Thus, the 90WBA-10G units have an apparent porosity of 26.3%. The addition of increasingly amounts of the calcium source, G, produces an increase in this property, rising to 42.0% for the 20WBA-80G specimens. Water absorption follows the same trend as apparent porosity, so the 90WBA-10G specimens have a water absorption of 15.6%, grows up to 30.28% for the 20WBA-80G units. As observed in the bulk density data, the increase in apparent porosity and water absorption of the calcium silicate units that incorporate between 40–50% of G is less pronounced, possibly due to the greater formation of hydraulics cementitious products, which are more compact. Water absorption values are similar to those obtained for calcium silicate units that use as raw materials coal fly ash and GeoSilex [43]; biomass bottom ash and calcium oxide or hydroxide calcium [38]. However, when Portland cement is used as a source of lime, lower water absorption values are obtained [38,42]. The maximum water absorption established by ASTMC67-07a: 2007 [61] depends on both the main purpose of calcium silicate units and the environment to which they are exposed. The maximum water absorption is 17% and 22%, respectively, depending on whether the specimens are exposed to severe or moderate weather conditions. If calcium silicate units are not exposed to the weather, no limits are established. Therefore, only 90WBA-10G and 80WBA-20G units can be used in severe weather conditions. The 70WBA-30G, 60WBA-40G and 50WBA-50G units can be used in moderate weather conditions. The rest of calcium silicate units can only be used coated.

**Figure 10:**
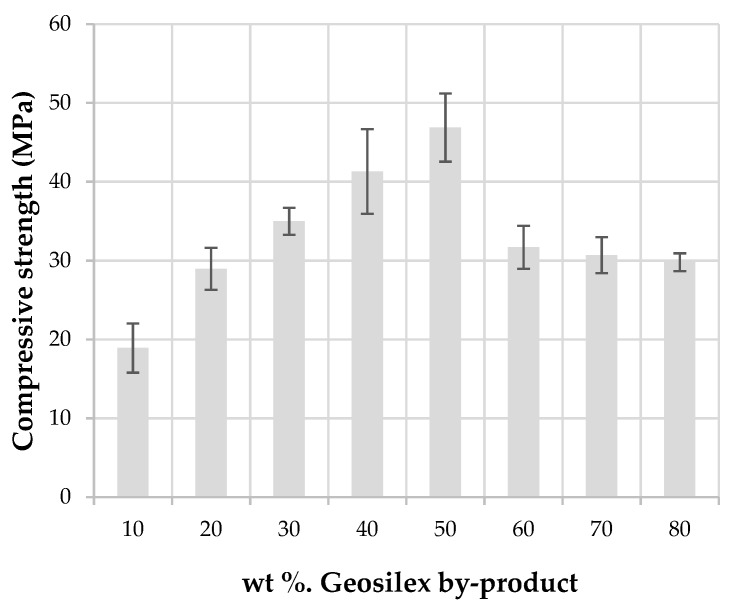
Compressive strength of the xWBA-yG calcium silicate units as function of G by-product content.

The compressive strength of building materials with structural function is the most important quality index in engineering. The mechanical strength in calcium silicate units is due to the formation of cementitious products in the pozzolanic reaction and during densification of the specimens. The compressive strength of the specimens (Figure 10) increases as the addition of GeoSilex to the WBA residue is increased up to 50 wt%. Thus, the calcium silicate units of 90WBA-10G have a compressive strength of 18.9 MPa, increasing for 50WBA-50G specimens up to 46.8 MPa. The best mechanical properties of this calcium silicate units may be due to the increased formation of calcium silicate hydrates (CSH), and calcium aluminate hydrates (CAH), responsible for the development of mechanical resistance, despite their greater open porosity as indicated by water absorption data. The incorporation of higher amounts of G by-product (60–80 wt%), produces a reduction in compressive strength. These specimens have a lower bulk density and greater apparent porosity and water absorption, and possibly the amount of WBA residue is insufficient and therefore the formation of calcium silicate hydrated to a lesser extent, which translates into lower mechanical properties. All units comply with EN 772-1: 2011 [49], which establishes a minimum compressive strength > 10MPa for use in masonry works.

The compressive strength obtained is similar to that obtained when coal fly ash and GeoSilex are used as raw materials [43]. However, higher values of compressive strength (66.6 MPa) are obtained when high contents of Portland cement (90 wt%) are used as a source of lime, with biomass bottom ash. Higher amounts of ash gives rise to calcium silicate units with lower compressive strength (29.5 MPa). The use of calcium hydroxide and calcium oxide gives rise to calcium silicate units with lower compression strength values between 12.3 and 28.6 MPa and between 14.3 and 36.8 MPa, respectively [38]. However, the use as raw materials as blast furnace slag and hydraulic lime gives rise to calcium silicate units with lower compressive strength (15.2 and 4.0 MPa) depending on the quicklime content and the forming pressure [39]. The use of bottom ashes from waste incineration or hospital solid waste incinerators used as raw materials with Portland cement leads to masonry units with compressive strength between 34.4 and 35.4 MPa after 28 days of curing, respectively [41]. The use of limestone powder and class C fly ash results in masonry units with compressive strengths of 15 MPa, increasing to 23 MPa with the addition of a 20 wt% of silica fume after 28 days of curing [40]. Therefore, the use of raw materials such as WBA and G gives rise to calcium silicate units with mechanical properties superior to those obtained when using other sources of lime, such as, calcium oxide, calcium hydroxide, quicklime and limestone. The mechanical properties obtained are similar to those obtained when Portland cement is used. However, it should be emphasized that the calcium silicate units obtained in this work are more sustainable by using GeoSilex as a source of lime, a by-product with almost no energy or environmental cost.

**Figure 11:**
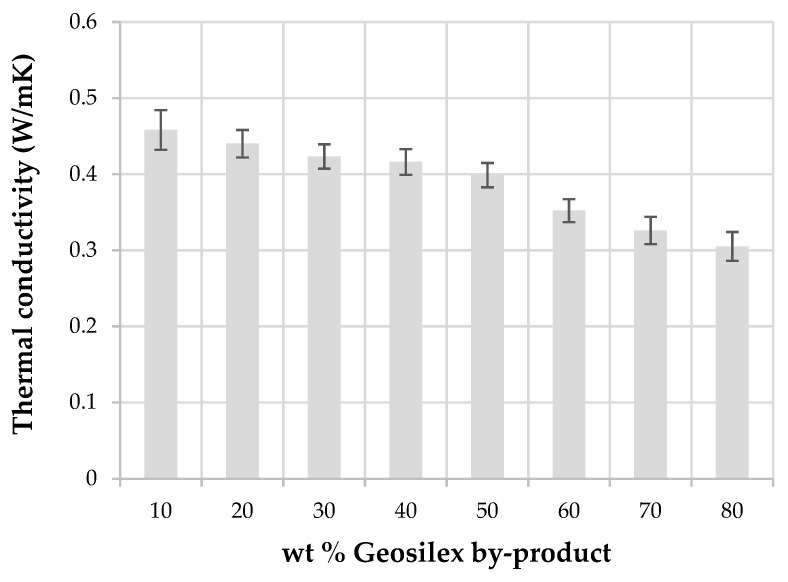
Thermal conductivity of the xWBA-yG calcium silicate units as function of G by-product content.

Thermal conductivity of calcium silicate units at 10 °C (Figure 11) indicates that thermal conductivity decreases as increasing amounts of G by-product are incorporated into the WBA residue. The thermal conductivity of 90WBA-10G units is 0.42 W/mK, decreasing to 0.30 W/mK for 20WBA-80G specimens. The thermal conductivity of specimens decreases as the bulk density of the calcium silicate units decreases, since bulk density is the main factor influencing the thermal conductivity of solid materials [62,63]. Calcium silicate units with low bulk density have a higher percentage of pores that are filled with air that acts as thermal insulator, increasing the insulation capacity of the solid matrix.

Thermal conductivity of conventional calcium silicate bricks is 0.8 W/mK. The conductivity values obtained for the xWBA-yG silica calcareous units are lower than those obtained when other alternative raw materials are used, such as, biomass bottom ash and calcium oxide (0.564 W/m K) or calcium hydroxide or Portland cement (0.773 and 0.785 W/mK, respectively) [38]; coal fly ash and limestone (0.99 W/mk). The addition of silica fume decreases thermal conductivity to 0.88 W/mK [40]. Therefore, using alternative raw materials to manufacture calcium silicate units such as WBA residue and G by-product can be achieved specimens with a better thermal insulation capacity.

**Figure 12:**
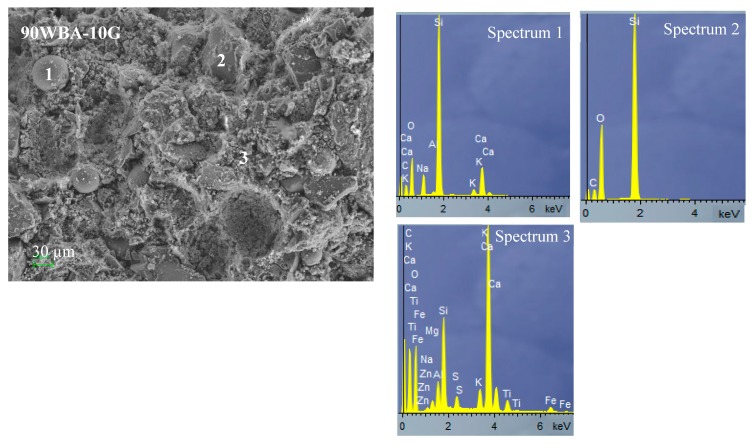

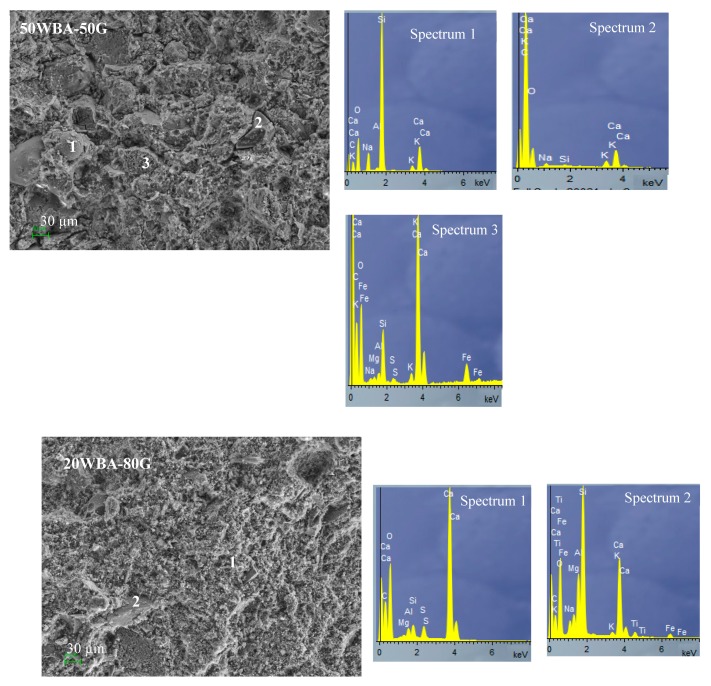
SEM-EDS analysis of 90WBA-10G, 50WBA-50G and 20WBA-80G calcium silicate units.

SEM microscopy and EDS analysis of calcium silicate units (90WBA-10G; 50WBA-50G and 20WBA-80G) (Figure 12) analyzed the structural cohesion as well as the distribution of porous structures. In all micrographs the quite amorphous structure formed by hydrated calcium silicates (CHS gel) is observed. The amount of gel formed is greater in the 50WBA-50G specimens according to the FTIR data. In addition, a greater amount of unreacted ash can be observed in the 90WBA-10G samples, due to the CaOH deficit. While in the 20WBA-80G units, unreacted CaOH particles are observed, due to the insufficient amount of SiO_2_. In the 50WBA-50G samples, spheres are also observed due to unreacted ashes and CaOH particles; however, the amount of raw materials without a reaction is lower.

## 4. Conclusions

In this work the possibility of manufacturing calcium silicate units using alternative raw materials has been studied, such as wood bottom ash as pozzolan and a by-product of the acetylene industry, GeoSilex, as a source of lime. The incorporation of increasing amounts of G by-product into the WBA residue results in calcium silicate units with lower bulk density and thermal conductivity. and higher apparent porosity and water absorption than conventional units. Compressive strength increases up to 50 wt% of G by-product is incorporated, resulting in a greater GeoSilex additions and a decrease in its mechanical property. The calcium silicate units with a 40–50 wt% of G by-product have an adequate amount of calcium hydroxide provided by the GeoSilex, and to a lesser extent by the ashes and pozzolanic materials (SiO_2_) present in the ashes, which give rise to a greater amount of hydrated calcium silicates responsible for the mechanical strength of the specimens. However, all calcium silicate units manufactured comply with the technical qualities required by the UNE-EN 771-2: 2011 standard for use in masonry work. Therefore, depending on the technological characteristics required of the specimens, different proportions of the raw materials may be used to achieve the desired properties. Therefore, it is possible to successfully value wood bottom ashes as a pozzolanic material and GeoSilex, a by-product, with a much lower energy and environmental cost than calcium oxide or Portland cement, to obtain sustainable calcium silicate units with adequate physical, mechanical and thermal properties.

## Figures and Tables

**Table 1:** Designation of calcium silicate units and raw materials, wood bottom ash, GeoSilex and water content of ten specimens.

Sample	WBA(g)	G(g)	Water (g)	WBA wt%	G wt%	Water wt% Total
90WBA-10G	360	40	50	90	10	12.5
80WBA-20G	320	80	48	80	20	12.0
70WBA-30G	280	120	44	70	30	11.0
60WBA-40G	240	160	40	60	40	10.0
50WBA-50G	200	200	38	50	50	9.5
40WBA-60G	160	240	36	40	60	9.0
30WBA-70G	120	280	34	30	70	8.5
20WBA-80G	80	320	32	20	80	8.0

**Table 2:** Organic matter, carbonate content, pH, specific surface area and relative density of wood bottom ash (WBA) and GeoSilex (G).

Raw Material	Organic Matter ^(a)^ (%)	Carbonate Content (%)	pH	Specific Surface Area (cm^2^/g)	Relative Density (kg/m^3^)
WBA	10.41 ± 0.09	17.25 ± 0.76	11.1	3600	2731
G	3.12 ± 0.12	16.6 ±0.55	12.5	6224	2378

^(a)^ Determined in accordance with ASTM D-2974.

**Table 3:** Chemical composition of wood bottom ash (WBA) and GeoSilex (G) raw materials.

Oxide Content (%)	WBA	G
SiO_2_	48.6	1.9
Al_2_O_3_	5.9	1.1
Fe_2_O_3_	3.3	0.12
CaO	18.1	67.2
MgO	3.2	0.09
MnO	0.05	-
Na_2_O	0.92	-
K_2_O	1.9	-
TiO_2_	1.4	0.04
P_2_O_5_	0.5	0.01
SO_3_	0.14	1.6
ZnO	0.29	-
SrO	0.04	-
Cl	0.06	0.03
LOI	15.6	27.8
